# Editorial: Pathogenic mechanisms, injury biomarkers, prophylaxis and treatment strategy of drug-induced nephrotoxicity

**DOI:** 10.3389/fmed.2024.1412795

**Published:** 2024-04-29

**Authors:** Guozheng Jiao, Yimin Niu, Bin Wang

**Affiliations:** ^1^Chemistry and Chemical Engineering Institute, Taishan University, Tai'an, Shandong, China; ^2^Department of Pharmacy, Zhongda Hospital, Southeast University, Nanjing, Jiangsu, China; ^3^Institute of Nephrology, Zhongda Hospital, Southeast University, Nanjing, Jiangsu, China

**Keywords:** drug-induced nephrotoxicity, pathogenic mechanisms, injury biomarkers, prophylaxis and treatment strategy, kidney, thioredoxin-interacting protein (TXNIP)

## Abstract

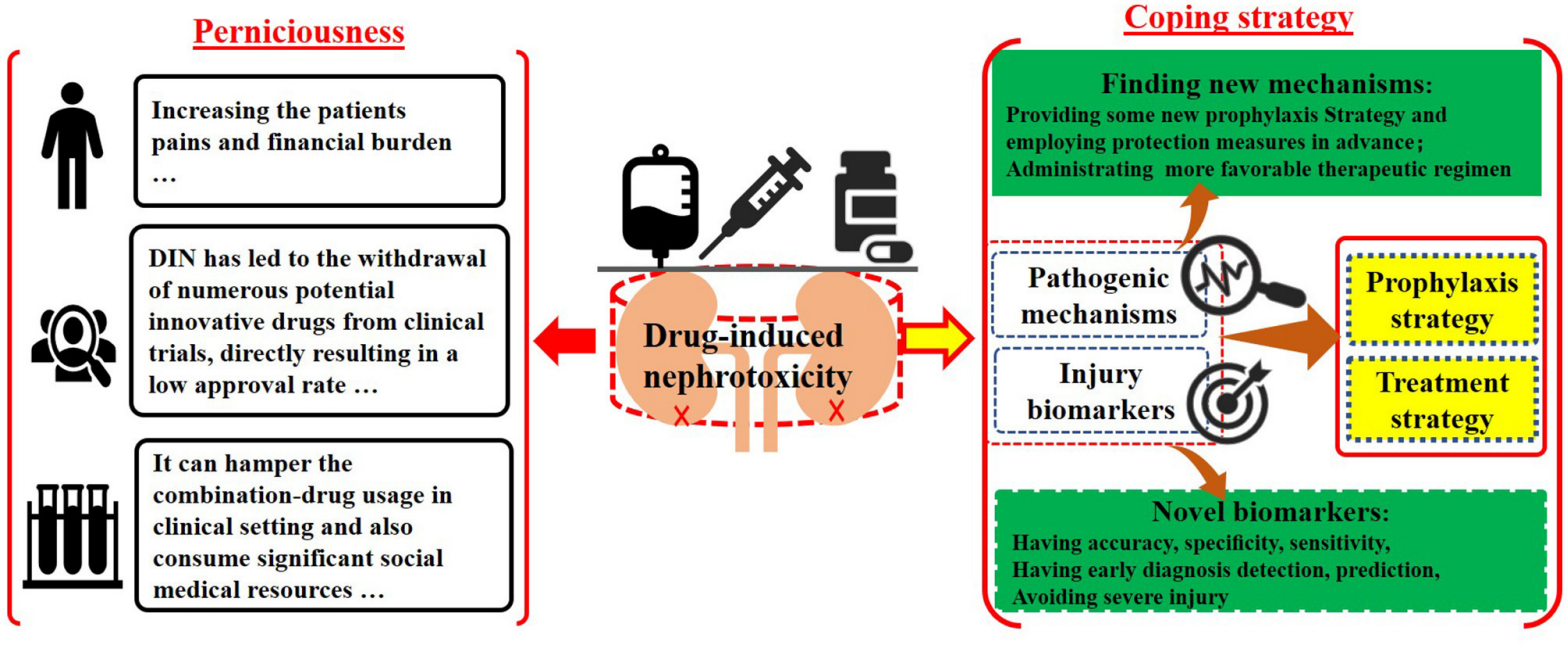

Drug-induced nephrotoxicity (DIN) exhibits high morbidity rate among global adverse reactions, posing a long-term potential burden on patients and society (1). In the United State, the annual number of DIN sufferers exceeds 1.5 million (2). Furthermore, in special groups such as older patients, and those with comorbid conditions, the incidence of drug-associated kidney damage is estimated to be increased further (1, 3, 4). Additionally, in intensive care unit (ICU), DIN events is not infrequently encountered (5). However, in specific clinical settings, such as those involving antiviral, antibacterial, and antitumor drugs (5), nephrotoxic agents may be necessary in the absence of alternative or supplementary agents (6). This may lead to potential kidney damage, which can worsen without adequate protective measures, ultimately resulting in kidney failure (7). Previous researches have indicated that DIN has led to the withdrawal of numerous potential innovative drugs from clinical trials, directly resulting in a low approval rate. Consequently, DIN has significantly hindered the research and development of new drug since healthy kidney tissue plays a crucial role in maintaining water-electrolyte balance, acid-base homeostasis, and overall physiological stability (2, 5). In clinical practice, apart from conventional tests such as serum creatinine (SCr), blood urea nitrogen (BUN) (6), and ultrasonography, there is a lack of highly sensitive and specific analytical methods currently available to assess kidney injury (8). When nephrotoxicity from various sources, including single or combination drug therapies, is identified, limited options exist beyond dosage adjustments or discontinuation (3).

The irreversible kidney damage caused by DIN presents a significant challenge, leading to substantial economic burdens, physical suffering for patients, restrictions on medication use, delays in drug development, and extensive utilization of healthcare resources. Consequently, DIN remains a pressing global health issue that warrants further investigation and understanding.

The Research Topic group aims to address the adverse effects of DIN by urging researchers to investigate two key factors: potential pathogenic mechanisms and kidney injury biomarkers, and to correlate them with prophylaxis and treatment strategies (Graphical Abstract). Understanding pathogenic mechanisms can reveal fundamental principles of kidney damage and guide potential preventive and therapeutic measures. By delving deeper into nephrotoxicity-related theoretical frameworks, researchers may identify specific components beyond the cellular level, which play significant roles in nephron damage, such as related to organelles, ligands, or second-messenger molecules. Additionally, there is an interest in uncovering how potential nephrotoxins, including drugs, herbs, nanomaterials, complexes, and metal ions, disrupt specific action receptors and elucidating the characteristics of their toxic effects (9). Therefore, researchers are encouraged to utilize cutting-edge technologies such as frozen electron microscopy, advanced imaging techniques, stem cell methodologies, and membrane potential analysis to develop targeted therapeutic interventions based on these specific mechanisms. With the advancement of analytical techniques such as high-resolution magic angle rotating proton NMR spectroscopy, multi-omics approaches, and bioinformatics-related molecular biology, a comprehensive multi-platform testing strategy can be systematically integrated. This integration enables the accurate identification of potential injury biomarkers by precisely tracking minute changes in quality and quantity of trace biomolecules. Traditional biomarkers like BUN, SCr, and urine protein, which are measured using less sensitive and specific instruments, often fail to detect abnormalities until significant damage has occurred, potentially leading to irreversible conditions. In contrast, advanced kidney injury biomarkers offer higher specificity, accuracy, and sensitivity, providing more comprehensive data. Early detection of specific biomarker traits before significant kidney damage occurs can significantly reduce the incidence of kidney injury, as timely interventions can be implemented. Furthermore, integrating advanced analytical results with bioinformatics and stem cell repair techniques can greatly enhance treatment strategies for kidney injuries. Early diagnosis, detection, and treatment of kidney injuries can reduce patient suffering, medical costs, and emotional distress, ultimately lowering mortality and morbidity rates.

Therefore, our group has conceptualized and designed a Research Topic focusing on advancing treatment strategies for DIN, with emphasis on understanding pathogenic mechanisms, identifying injury biomarkers, and developing prophylactic measures. Sun et al. highlighted the role of thioredoxin-interacting protein (TXNIP) in diabetic kidney damage (DKD), noting its upregulation in DKD patients compared to healthy individuals. They suggested TXNIP as a promising therapeutic target. Wang et al. investigated the association between elevated uric acid levels and gouty nephropathy (GN) and proposed relevant animal models that mirror clinical manifestations. By exploring response mechanisms, they identified potential treatment targets for various kidney injuries. Zhou et al. not only reviewed mechanisms of DIN injury but also explored the correlation between DIN and urinary exosomes as potential biomarkers for accurate clinical testing. Zou et al. utilized a mathematical model to assess the therapeutic efficacy of a combination medicine containing sirolimus (SRL) and low-dose extended-release tacrolimus (LER-TAC) in kidney transplant patients. Their findings indicated superior efficacy and safety of the combination therapy, suggesting a promising treatment strategy for kidney transplant recipients, especially those requiring immunosuppression.

## Author contributions

GJ: Conceptualization, Supervision, Writing – original draft, Writing – review & editing. YN: Conceptualization, Formal analysis, Investigation, Writing – review & editing, Supervision. BW: Conceptualization, Investigation, Writing – review & editing.
